# *Lactobacillus crispatus* inhibits the infectivity of *Chlamydia trachomatis* elementary bodies, in vitro study

**DOI:** 10.1038/srep29024

**Published:** 2016-06-29

**Authors:** Paola Nardini, Rogers Alberto Ñahui Palomino, Carola Parolin, Luca Laghi, Claudio Foschi, Roberto Cevenini, Beatrice Vitali, Antonella Marangoni

**Affiliations:** 1 Microbiology, DIMES, University of Bologna, Via Massarenti, 9, 40138, Bologna, Italy; 2 Department of Pharmacy and Biotechnology, University of Bologna, Via San Donato, 19/2, 40127 Bologna, Italy; 3 Centre of Foodomics, Department of Agro-Food Science and Technology, University of Bologna, P.za Goidanich, 60, 47521, Cesena, Italy

## Abstract

*Lactobacillus* species dominate the vaginal microbiota of healthy reproductive-age women and protect the genitourinary tract from the attack of several infectious agents. *Chlamydia trachomatis*, a leading cause of sexually transmitted disease worldwide, can induce severe sequelae, i.e. pelvic inflammatory disease, infertility and ectopic pregnancy. In the present study we investigated the interference of *Lactobacillus crispatus*, *L. gasseri* and *L. vaginalis*, known to be dominant species in the vaginal microbiome, with the infection process of *C. trachomatis*. Lactobacilli exerted a strong inhibitory effect on *Chlamydia* infectivity mainly through the action of secreted metabolites in a concentration/pH dependent mode. Short contact times were the most effective in the inhibition, suggesting a protective role of lactobacilli in the early steps of *Chlamydia* infection. The best anti-*Chlamydia* profile was shown by *L. crispatus* species. In order to delineate metabolic profiles related to anti-*Chlamydia* activity, *Lactobacillus* supernatants were analysed by ^1^H-NMR. Production of lactate and acidification of the vaginal environment seemed to be crucial for the activity, in addition to the consumption of the carbonate source represented by glucose. The main conclusion of this study is that high concentrations of *L. crispatus* inhibit infectivity of *C. trachomatis in vitro*.

The health and functioning of the female urogenital tract largely rely on its microbial inhabitants^1^. The normal microbiota of the human vagina plays a key role in preventing a number of urogenital diseases, such as bacterial vaginosis, yeast infections, urinary tract infections and sexually transmitted infections^2^,^3^,^4^,^5^,^6^,^7^. These protective functions are mainly attributed to *Lactobacillus* species that dominate the vaginal niche of healthy women^8^. Lactobacilli play key protective roles through different mechanisms: production of various antibacterial compounds (lactic acid, hydrogen peroxide, bacteriocins and biosurfactants), co-aggregation, competitive exclusion, immunomodulation, and signalling between bacteria that can lead to down-regulation of toxin production in pathogens^9^,^10^.

Extending the concept of lactobacilli as endogenous defence factors, there is an increasing interest for probiotics in the context of urogenital health^11^. Lactobacilli have been proposed as agents for the prevention and treatment of urinary tract infections^12^, bacterial vaginosis^13^ and even for the prevention of HIV and sexually transmitted infections^14^.

The obligate intracellular bacterium *Chlamydia trachomatis* is a leading cause of sexually transmitted infections (STIs) with more than 100 million new cases per year according to global estimates^15^. A high proportion of chlamydial STIs are asymptomatic and thereby left untreated, favouring both the transmission and the occurrence of serious complications like pelvic inflammatory disease, infertility, ectopic pregnancies and preterm deliveries^16^,^17^,^18^,^19^*. C. trachomatis* has a unique cycle of development, alternating between two distinct bacterial forms. The elementary body (EB) is infectious but non-dividing. In contrast, the reticulate body (RB) is non-infectious but replicative^20^. After attachment and penetration in cells, EBs remain internalized in vacuoles that escape phago-lysosomal fusion. Within these vacuoles, named inclusions, EBs differentiate into RBs after several transformations. Unlike EBs, RBs are larger, less compacted, metabolically active and able to divide by binary fission. In *C. trachomatis*, around 18 h post-infection, RBs resulting by binary fission differentiate back into EBs that will afterwards be expelled from the cell, either by exocytosis or cellular lysis 48–72 h post-infection^21^.

In spite of the importance of a healthy vaginal microbiota in preventing genital infections, only a few studies have focused on the protective effects of vaginal lactobacilli towards chlamydial infection^22^,^23^,^24^. Adverse effects by lactobacilli on chlamydial EBs, chlamydial absorption to epithelial cells and intracellular phases of chlamydial replication have been demonstrated. However, the molecular mechanisms underlying the interactions between *Lactobacillus* and *C. trachomatis* in the vaginal environment have not yet been elucidated.

The aim of the present work was to investigate the impact of seventeen *Lactobacillus* strains isolated from the vagina of healthy women on infectivity of *C. trachomatis* EBs against HeLa cells, as well as to identify metabolic profiles related to the antibacterial activity. The identification of *Lactobacillus* strains and/or metabolites active against *C. trachomatis* is the first step of a broader research project aimed at identifying new probiotic strategies to prevent a sexually transmitted infection that adversely affects women’s health.

## Results

### Effects of lactobacilli cell free supernatants on *C. trachomatis* infectivity

In order to investigate the potential antagonist role of vaginal lactobacilli against *C. trachomatis*, we evaluated the ability of seventeen *Lactobacillus* strains to inactivate infectivity of EBs, before they interact with cellular host receptors. These lactobacilli were previously isolated from vaginal swabs of healthy premenopausal women^25^ and belong to three species highly represented in the vaginal habitat: *Lactobacillus crispatus* (BC1-BC8), *Lactobacillus gasseri* (BC9-BC14) and *Lactobacillus vaginalis* (BC15-BC17) (Table 1).

**Figure 1:**
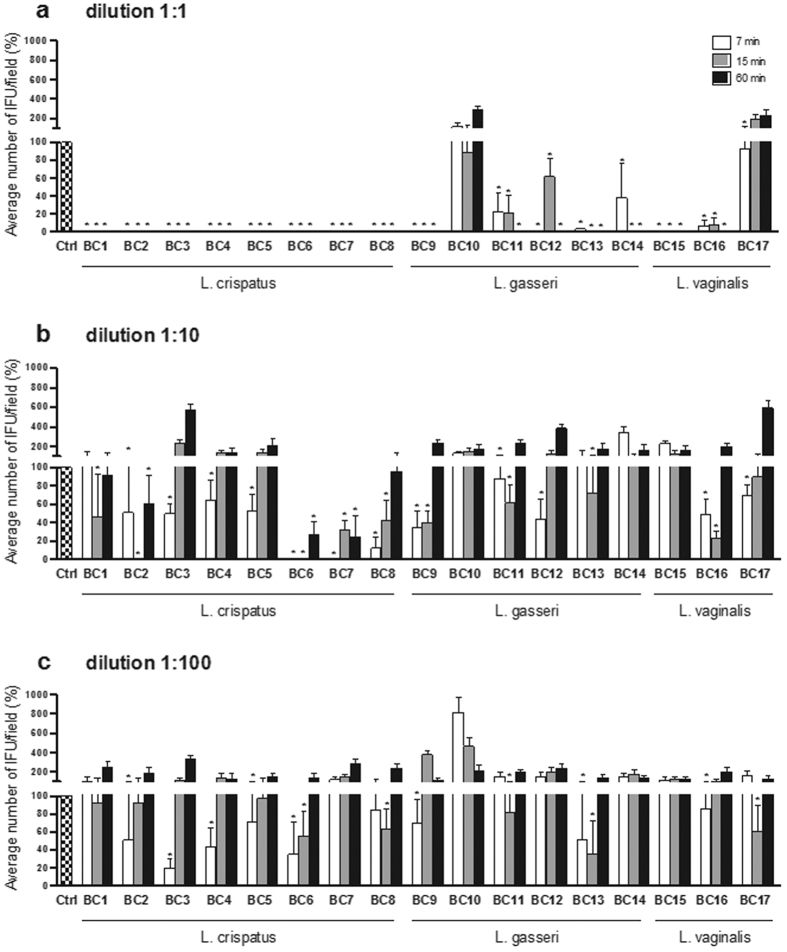
Effect of lactobacilli supernatants on *C. trachomatis* infectivity. Experiments were performed with different dilutions of cell free supernatants: 1:1 (**a**), 1:10 (**b**) and 1:100 (**c**), and different time points: 7 minutes (white bars), 15 minutes (grey bars) and 60 minutes (black bars). *C. trachomatis* infectivity was evaluated as number of IFU/microscopic field. The results were expressed in percentage compared with control, taken as 100% (dotted bars). Bars represent median values, error bars represent median absolute deviations. Statistical significance was calculated vs control. *P < 0.05.

Three different dilutions (1:1; 1:10; 1:100) of lactobacilli cell free supernatants (CFS) were tested at three different time points (7, 15, 60 min). After the challenge with *Lactobacillus* CFS, the capacity of *C. trachomatis* EBs to infect HeLa cells was assessed by immunofluorescence. EBs infectivity was expressed in terms of percentage of inclusions forming units (IFU)/field (median  ±  median absolute deviation) compared to control (Fig. 1).

At the highest supernatant concentration (dilution 1:1), the majority of *Lactobacillus* strains significantly reduced the infectivity of *C. trachomatis* EBs against HeLa cells. Ten *Lactobacillus* strains (*L. crispatus* BC1-BC8, *L. gasseri* BC9 and *L. vaginalis* BC15) completely abolished the infectivity of *Chlamydia* EBs at any time point. The supernatants of five *Lactobacillus* strains (*L. gasseri* BC11-BC14 and *L. vaginalis* BC16) decreased *C. trachomatis* infectivity at any contact time, with a complete inhibition after a long term exposure (60 min). *L. vaginalis* BC17 showed a moderate anti-*Chlamydia* activity at short contact time (7 min) and *L. gasseri* BC10 did not exert any inhibitory activity (Fig. 1a). *L. crispatus* BC2, BC6 and BC7 supernatants diluted 1:10 were still capable of significantly reducing *C. trachomatis* infectivity at all three time points. *L. crispatus* BC1, BC3-BC5 and BC8, *L. gasseri* BC9, BC11-BC13, and *L. vaginalis* BC16 and BC17 retained the anti-*Chlamydia* activity at short time points (7 and/or 15 min). *Lactobacillus gasseri* BC10 and BC14, and *L. vaginalis* BC15 CFS did not alter *Chlamydia* EBs (Fig. 1b). At the lowest concentration (dilution 1:100), eleven *Lactobacillus* strains (*L. crispatus* BC2-BC6 and BC8, *L. gasseri* BC9, BC11 and BC13, and *L. vaginalis* BC16 and BC17) decreased *C. trachomatis* infectivity when applied for short contact times, while no *Lactobacillus* strain was effective after 60 minutes of exposure. At the lowest concentration, *L. crispatus* BC1 and BC7, and *L. gasseri* BC12 supernatants did not exert any inhibitory activity against *C. trachomatis* EBs (Fig. 1c).

In general, *L. crispatus* supernatants were the most powerful in counteracting *C. trachomatis* infectivity, as all of them abrogated *Chlamydia* inclusions at the highest concentration (1:1), and retained the greatest reductions at the intermediate (1:10) and lowest (1:100) concentrations, especially BC6 and BC8 strains. Six strains (BC2-BC6, BC8) out of eight belonging to *L. crispatus* species maintained a good activity even at 1:100 concentration; indeed, they caused a significant reduction of *Chlamydia* infectivity for at least one time point. Among *L. gasseri* and *L. vaginalis* strains, heterogeneous activity profiles have been found, especially at the highest concentration: *L. gasseri* BC9 and *L. vaginalis* BC15 and BC16 supernatants were very effective in *C. trachomatis* inhibition, in contrast to *L. gasseri* BC10 and *L. vaginalis* BC17.

The inhibitory activity of lactobacilli supernatants towards *C. trachomatis* was strictly concentration-dependent, being fifteen CFS (out of seventeen) effective in reducing EBs infectivity at the highest concentration, whereas only eleven retained a certain efficacy after 1:100 dilution. Notably, at the highest concentration, lactobacilli culture supernatants were found to have pH values comprised in the range 3.71-5.28 (pH mean value 4.19 ± 0.42). On the contrary, diluted lactobacilli supernatants showed higher pH values, in the range 4.3–7.15 (pH mean value 5.84 ± 0.86) for the dilution 1:10, and in the range 6.76–7.31 (pH mean value 7.08 ± 0.15) for the dilution 1:100 (Supplementary Table S1). This finding indicated a strict link between acidity and the ability to inactivate *Chlamydia* EBs. Indeed, pH values of culture supernatants were positively correlated with *Chlamydia* IFU/field median values, showing a Spearman coefficient of 0.7486 (two-tailed P value = 9.7357 × 10^−29^). Moreover, both at the intermediate and lower concentration lactobacilli CFS exhibited higher efficacy when applied for short contact times (7 and/or 15 min).

### Effects of lactobacilli cell pellets on *C. trachomatis* infectivity

**Figure 2:**
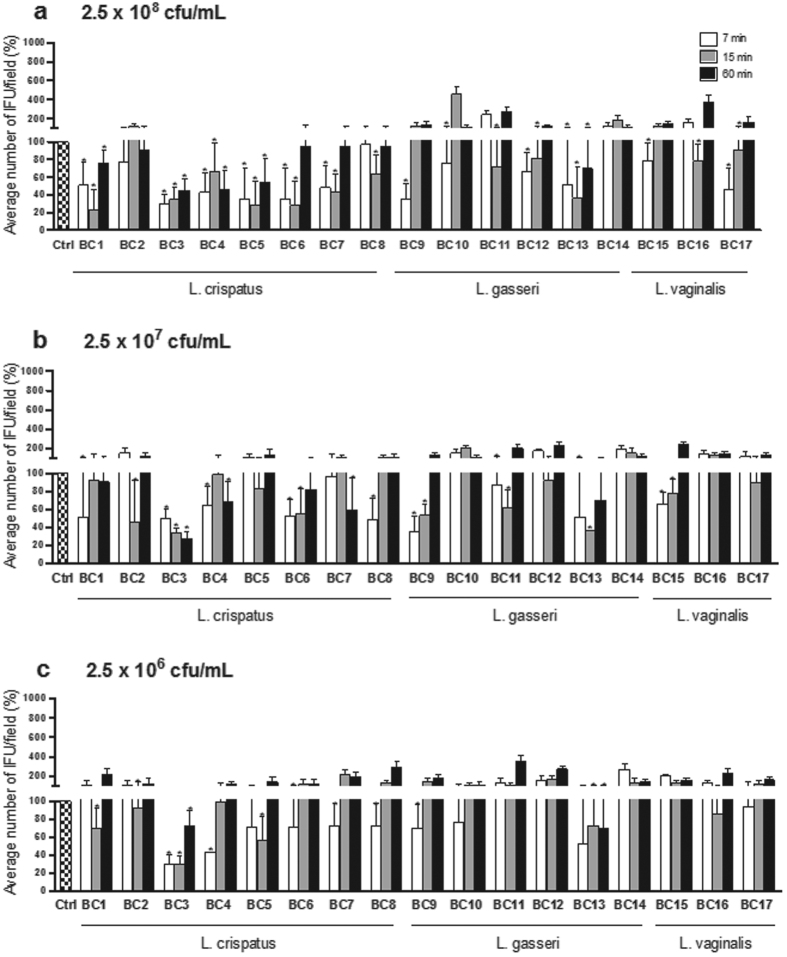
Effect of lactobacilli cell pellets on *C. trachomatis* infectivity. Experiments were performed at different concentrations of cell pellets: 2.5 × 10^8^ CFU/mL (**a**), 2.5 × 10^7^ CFU/mL (**b**) and 2.5 × 10^6^ CFU/mL (**c**), and different time points: 7 minutes (white bars), 15 minutes (grey bars) and 60 minutes (black bars). *C. trachomatis* infectivity was evaluated as number of IFU/microscopic field. The results were expressed in percentage compared with control, taken as 100% (dotted bars). Bars represent median values, error bars represent median absolute deviations. Statistical significance was calculated vs control. *P < 0.05.

Similarly to CFS, lactobacilli cell pellets (CP) were tested at three different concentrations (2.5 × 10^8^, 2.5 × 10^7^ and 2.5 × 10^6^ CFU/mL) and time points (7, 15, 60 min). The results are expressed in terms of percentage of *C. trachomatis* IFU/field (median ± median absolute deviation) compared to control (Fig. 2).

At the concentration of 2.5 × 10^8^ CFU/mL, five *Lactobacillus* cell pellets (*L. crispatus* BC1, BC3-BC5 and *L. gasseri* BC13) strongly reduced *C. trachomatis* infectivity at any time point. For short contact times (7 and/or 15 min) a significant inhibitory activity was observed for ten lactobacilli (*L. crispatus* BC6-BC8, *L. gasseri* BC9-BC12 and *L. vaginalis* BC15-BC17). *L. crispatus* BC2 and *L. gasseri* BC14 cells did not affect *C. trachomatis* EBs infectivity (Fig. 2a). At the intermediate concentration of lactobacilli cell pellets (2.5 × 10^7^ CFU/mL), only *L. crispatus* BC3 retained a strong inhibitory activity at any time point. *L. crispatus* BC1, BC2, BC4, BC6, BC8 and *L. gasseri* BC9, BC11, BC13, and *L. vaginalis* BC15 significantly reduced *C. trachomatis* infectivity at short exposure times, while *L. crispatus* BC7 showed activity after 60 minutes of contact. No inhibitory effect was exerted by *L. crispatus* BC5, *L. gasseri* BC10, BC12, BC14, and *L. vaginalis* BC16 and BC17 (Fig. 2b). At the concentration of 2.5 × 10^6^ CFU/mL, *L. crispatus* BC3 cells were still able to inhibit *C. trachomatis* infectivity at all contact times, and *L. crispatus* BC1, BC2, BC4-BC8 and *L. gasseri* BC9 and BC13 were effective at short time points. *Lactobacillus gasseri* BC10-BC12 and BC14 and *L. vaginalis* BC15-BC17 cells did not show any inhibitory effect (Fig. 2c).

In analogy with the results obtained using the supernatants, *L. crispatus* cells were the most effective in reducing *C. trachomatis* infectivity, exhibiting good inhibitory skills at the concentration of 2.5 × 10^8^ CFU/mL, and being almost all active after dilution for at least one time point. *L. gasseri* and *L. vaginalis* cell pellets showed a more concentration-dependent activity, since their dilution caused the loss of *Chlamydia* inhibition for four *L. gasseri* strains (out of six) and for all *L. vaginalis* strains. Moreover, the challenge experiments with lactobacilli cells confirmed the major efficacy for short contact times (7 and/or 15 min), independently of the cell concentration.

### *C. trachomatis* inhibition by lactic acid and hydrochloric acid

**Figure 3:**
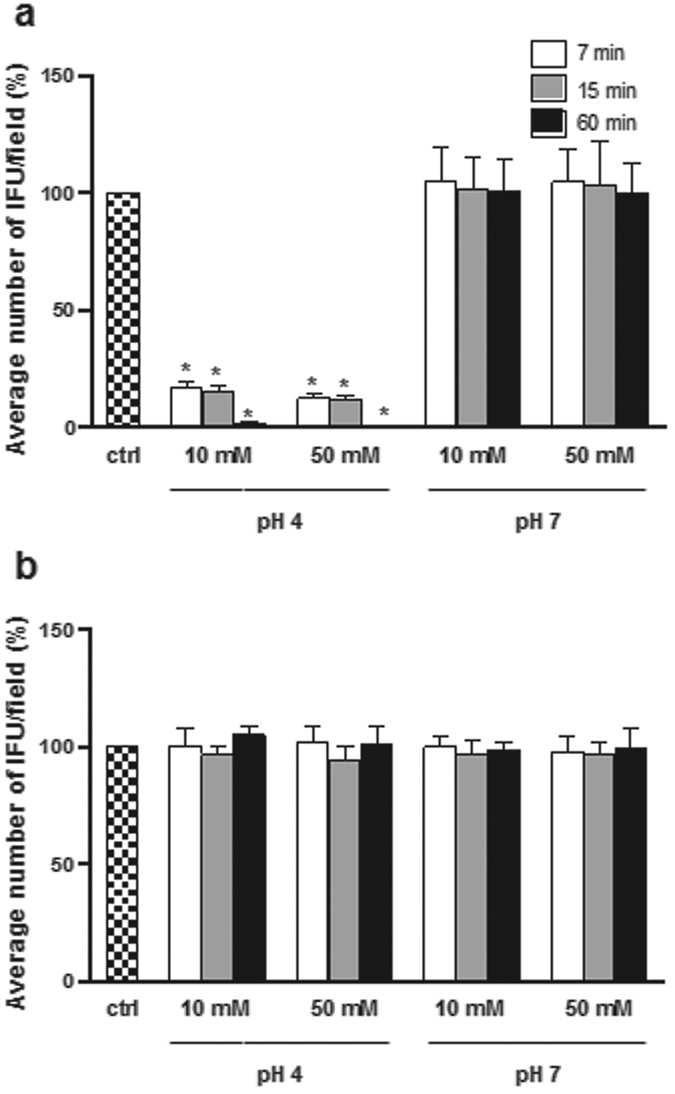
Effect of lactic acid and hydrochloric acid on *C. trachomatis* infectivity. Experiments were performed with lactic acid (**a**) and HCl (**b**). Infectivity was evaluated for different concentrations of lactic acid/HCl (10 mM and 50 mM), pH values (4 and 7), and time points [7 minutes (white bars), 15 minutes (grey bars) and 60minutes (black bars)]. *C. trachomatis* infectivity was evaluated as number of IFU/microscopic field. The results were expressed in percentage compared with control, taken as 100% (dotted bars). Bars represent median values, error bars represent median absolute deviations. Statistical significance was calculated vs control. *P < 0.05.

The effect of lactic acid on *C. trachomatis* infectivity was evaluated after challenge of EBs for 7, 15 and 60 min. Two concentrations of lactic acid (10 mM and 50 mM), corresponding to the mean titer and the highest titer registered by ^1^H-NMR in *Lactobacillus* CFS (Supplementary Table S2), were tested. Lactic acid solutions were buffered at two pH values (pH 4 and pH 7), corresponding to the pH range registered in *Lactobacillus* CFS (Supplementary Table S1) and physiologically found in the vagina. The inhibitory activity of hydrochloric acid (HCl) on *C. trachomatis* infectivity was evaluated in the same experimental conditions (10 mM and 50 mM; pH 4 and pH 7) to compare the effects of lactic acid with those exerted by an inorganic acid. The results are expressed in terms of percentage of *Chlamydia* IFU/field (median ± median absolute deviation) compared to control (Fig. 3).

At acid pH (pH 4), lactic acid was able to strongly inhibit EBs infectivity, both at 10 and 50 mM, and for all exposure times. After 60 min of contact, *Chlamydia* infectivity was virtually completely lost. In contrast, lactic acid lost any chlamydiacidal activity when buffered at pH 7, regardless of contact time (Fig. 3a). HCl did not interfere with *Chlamydia* infectivity, neither at 10 mM nor at 50 mM, at any pH value (Fig. 3b). These results indicate that the presence of a high concentration of H^+^ ions is essential but not sufficient for the inhibition of *Chlamydia* EBs.

### Identification of *Lactobacillus* strains exerting anti-*Chlamydia* activity

With the aim to delineate a ranking of *Lactobacillus* strains on the basis of their capability to counteract *Chlamydia* infectivity, we approached a statistical analysis on the entire set of median values, considering any concentration and time point. As a first step, we wondered if lactobacilli CP and CFS fractions were equally effective in reducing *Chlamydia* infectivity. To address this question, we firstly compared all median data collected with *Chlamydia* EBs pre-incubated with CP fractions to data of untreated EBs, by means of a non-parametric statistical test: the infectivity of *Chlamydia* EBs pre-incubated with CP was not significantly different from the infectivity of untreated EBs (P = 0.4245, 1-tailed Wilcoxon signed rank test). Similarly, we compared median data obtained with *Chlamydia* EBs pre-incubated with CFS fractions to data of untreated EBs, and we ascertained that pre-incubation of EBs with CFS significantly reduced *Chlamydia* infectivity (P = 0.0384, 1-tailed Wilcoxon signed rank test). Indeed, comparing median data obtained with CP-treated EBs to those obtained with CFS-treated EBs, we confirmed that anti-*Chlamydia* effect of lactobacilli CFS were significantly different from that of the respective CP (P = 0.0043, 1-tailed Wilcoxon matched paired rank test).

**Figure 4:**
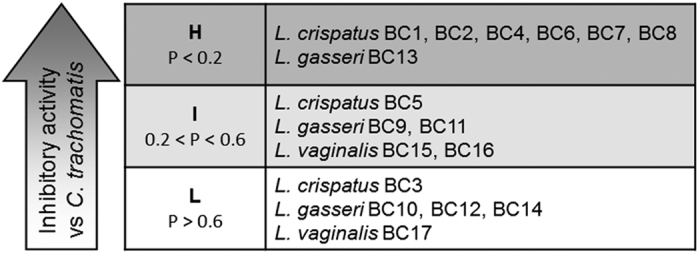
Ranking of lactobacilli in relation to anti-*Chlamydia* activity. *Lactobacillus* strains were classified on the basis of the inhibitory activity of their cell free supernatants (CFS), expressed as the difference between CFS-treated EBs and untreated EBs, by means of the 1-tailed Wilcoxon signed rank P-values. Group H comprises lactobacilli strains with P-values below 0.2, group I comprises of lactobacilli with P-values ranging between 0.2 and 0.6; group L comprises lactobacilli with P-values over 0.6.

Being CFS the fraction capable of reducing *Chlamydia* infectivity, we classified lactobacilli only on the basis of the anti-*Chlamydia* activity exerted by CFS. For each *Lactobacillus* strain, CFS efficacy has been expressed as the odds between data collected with CFS-treated EBs and untreated EBs (control, taken as 100%), by means of the 1-tailed Wilcoxon signed rank P-values (Supplementary Table S3). A low P-value indicates that medians obtained with *Chlamydia* EBs pre-incubated with *Lactobacillus* CFS are different from the control. Conversely, a high P-value denotes that medians obtained with *Chlamydia* EBs pre-treated with *Lactobacillus* CFS are similar to the control. Thus, we classified *Lactobacillus* strains into 3 groups in relation to the inhibitory activity (Fig. 4). The first group (H, high activity) consists of lactobacilli with P-values below 0.2, i.e. lactobacilli that perturb more *Chlamydia* infectivity; the second group (I, intermediate activity) consists of lactobacilli with P-values ranging between 0.2 and 0.6; the last group (L, low activity) comprises lactobacilli with P-values over 0.6, i.e. lactobacilli that are less effective toward *Chlamydia* infectivity. Group H counts 7 strains (BC1, BC2, BC4, BC6, BC7, BC8, and BC13), group I comprises 5 strains (BC5, BC9, BC11, BC15, and BC16), group L included 5 strains as well (BC3, BC10, BC12, BC14, and BC17).

### Identification of *Lactobacillus* metabolic profiles associated with anti-*Chlamydia* activity

Lactobacilli CFS were analyzed by ^1^H-NMR to delineate their metabolic profiles. We identified 40 molecules mainly belonging to the families of aminoacids, organic acids monosaccharides, ketones and alcohols.

**Figure 5:**
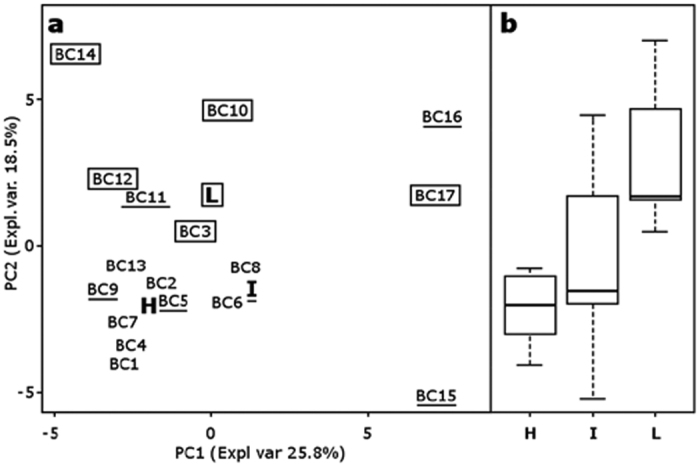
Correlation between metabolome of lactobacilli and inhibitory activity towards *C. trachomatis*. (**a**) Score plot of *Lactobacillus* strains on PC1 and PC2 of a PCA model built on the total metabolites identified by ^1^H-NMR in cell free supernatants. H, I and L indicate the median values of lactobacilli grouped according to anti-*Chlamydia* activity. Strains without marks belong to group H; underlined strains belong to group I; strains within rectangles belong to group L. Expl. Var, explained variance. (**b**) Box plots representing the distribution of activity against *Chlamydia* in relation to the metabolome. Lines within the boxes indicate the median values of the samples groups corresponding to the different activity scores (H: high activity; I: intermediate activity; L: low activity). Each box represents the interquartile range (25–75th percentile). The bottom and top bars indicate the 10th and 90th percentiles, respectively.

A Principal Component Analysis (PCA) model was built on the concentrations of the identified molecules in order to search for correlations between anti-*Chlamydia* activity and metabolome of latobacilli (Fig. 5). In the biplot describing the distribution of *Lactobacillus* strains in relation to the pool of metabolites, Principal Component (PC) 1 and PC2 accounted for the 44.3% of the whole variance of the investigated samples (Fig. 5a). The first component, accounting for the 25.8% of the total variance, was found to be mainly influenced by the taxonomy of lactobacilli, in agreement with Parolin *et al.*^25^. The second component, accounting for the 18.5% of the total variance, was found to be mainly influenced by the activity of lactobacilli against *Chlamydia*. The correlation between metabolome and anti-*Chlamydia* activity was best visualized by means of box plots representing the distribution of the groups of lactobacilli (H, I and L), created on the basis of their inhibitory activity against *Chlamydia* EBs, in relation to the metabolome (Fig. 5b). Strains with different anti-*Chlamydia* activity were clearly separated in the vertical direction: the most active strains occupied the lower positions while the less active strains were placed in the higher areas of the two-dimensional space. The highest metabolic homology was observed within the group of lactobacilli exerting high activity, as demonstrated by the lower height of the corresponding boxplot.

To gain information about the molecules which mainly determined the metabolome-activity link, the correlation between each molecule concentration change and its loading along PC2 was calculated, according to the approach of De Filippis *et al.*^26^. Seven molecules showed a correlation higher than 0.6 (Supplementary Table S2). Orotate was produced in greater concentrations by lactobacilli highly active against *Chlamydia* (group H) while phenylalanine, isoleucine, valine and tyrosine were produced in greater concentrations by lactobacilli less effective against *Chlamydia* (group L); glucose was consumed at higher levels by lactobacilli belonging to group H while tryptophan was consumed more by lactobacilli of group L. Orotate and phenylalanine production was found to be statistically different between lactobacilli in groups H and L (Orotate, P = 0.005; Phenylalanine, P = 0.005; 2-tailed Wilcoxon signed rank test). Inhibitory activity of orotic acid was evaluated, but no significant reduction of *C. trachomatis* infectivity was observed at the highest concentration found in the lactobacilli supernatants (30 μM) (Supplementary Figure S1a). Orotic acid was also tested in association with lactic acid (10/50 mM; pH: 4/7), but this combination did not lead to an enhancement of lactic acid effect (Supplementary Figure S1b). However, we cannot exclude a synergistic action of this metabolite in the more complex cultural medium where other bacterial molecules may act as enhancers. It is worth underlining the increased consumption of glucose by the strains of Group H. In order to understand whether the competition for the carbonate source could be an additional mechanism of action for the antagonism towards *Chlamydia*, further inhibition experiments were performed by adding glucose to *Lactobacillus* supernatants. *L. crispatus* BC1 and *L. gasseri* BC13 were selected as model strains because they consumed the highest amount of glucose within the H group (high activity). We added glucose at the concentration of 30 mM in order to reset the sugar consumption by these active lactobacilli. The addition of glucose to *L. crispatus* BC1 supernatant led to a significant increase (51 fold) of *C. trachomatis* infectivity at the shortest contact time (7 minutes), while no increase of infectivity was found at the time points 15 and 60 minutes. Similarly, *L. gasseri* BC13 supernatant enriched with glucose showed a reduction in anti-*Chlamydia* activity both after 7 minutes (infectivity increase: 8.7 fold) and 15 minutes (infectivity increase: 6.1 fold) of contact (Supplementary Table S4). These data confirm the importance of the depletion of glucose as an additional mechanism for the inhibition of *C. trachomatis* EBs by vaginal lactobacilli, in particular for short contact times.

## Discussion

*Chlamydia trachomatis* is the most common cause of bacterial sexually transmitted infections in both industrialized and developing countries^15^,^27^. Cross sectional and longitudinal studies have demonstrated a positive correlation between bacterial vaginosis, characterized by a lack of *Lactobacillus* species, and the incidence of sexually transmitted infections, including *C. trachomatis*^6^,^28^,^29^,^30^,^31^. Residing at the port of entry of bacterial and viral pathogens, the vaginal lactobacilli create a barrier against pathogen invasion since products of their metabolism secreted in the cervicovaginal fluid can play an important role in the inhibition of infectious agents^32^.

This *in vitro* study provides experimental evidence for inhibition of *C. trachomatis* infection by vaginal lactobacilli. It has been hypothesized that *Lactobacillus* species play a critical protective role in the vaginal niche by producing lactic acid, responsible for low pH, which inhibits sexually transmitted pathogens. Recently, Gong *et al.* suggested that low pH is fully responsible for chlamydiacidal activity of lactobacilli^22^.

The present *in vitro* study showed that *Lactobacillus* strains exert a strong inhibitory effect on *Chlamydia* infectivity mainly through the action of metabolites secreted out of the cell, in a concentration-dependent manner. Because organic acids are the principal metabolic products of lactobacilli, the factor “concentration” is inversely correlated to the pH of the culture medium. Indeed, cell-free supernatants corresponding to the highest lactobacilli concentration were characterized by low pH, while diluted lactobacilli supernatants showed higher pH.

The factor “contact time” also seems to play a role in the inhibitory activity. Lactobacilli, especially as regards the cellular fractions, were more effective in inhibiting *Chlamydia* EBs infectivity when applied for short contact times. The inhibition may be generated by a rapid and dynamic modification of EBs membrane induced by the interaction with lactobacilli; we assume that this modification could revert for longer exposure times. The finding of this *in vitro* study suggests that lactobacilli could exert their protective role against *Chlamydia* in the early steps of the infection, probably due to inactivation of EBs before they can colonize and infect the host hepitelial cells, according to results previously described^23^.

In order to interpret rationally the wealth of data produced in this study, we used an approach that allowed us to classify *Lactobacillus* strains into three groups (H: high activity; I: intermediate activity; L: low activity) according to their ability to counteract *Chlamydia* infectivity. The best anti-*Chlamydia* profile was shown by strains belonging to *L. crispatus* species. Conversely, *L. gasseri* and *L. vaginalis* showed a heterogeneous spectrum of activity against *C. trachomatis*. A similar correlation between biological activity and taxonomy has recently been reported in relation to the antagonism exerted by the same *Lactobacillus* strains towards *Candida* infection^25^.

*L. crispatus* promotes the stability of the normal vaginal microbiota^32^,^33^, and also seems to have a role in the restoration of the vaginal communities, and in the maintenance of remission from bacterial vaginosis, following antibiotic treatment^34^. It has been reported that strains of *L. crispatus* inhibit *in vitro* the growth of uropathogens and block their adhesion to vaginal epithelial cells^35^. *L. crispatus* was also shown to reduce the adhesion of *Neisseria gonorrhoeae*^36^ and *C. trachomatis*^23^ to HeLa cells by competitive exclusion of pathogenic species. Moreover, recent data demonstrate the efficacy of *L. crispatus* to limit the inflammatory reaction in *C. trachomatis*-infected HeLa cells and macrophages^24^.

Directly linked to the presence of lactobacilli, the production of lactic acid is accepted as a hallmark beneficial activity of the vaginal microbiota. Lactic acid has been associated with pathogen exclusion and its concentration could also be seen as an important biomarker of vaginal health, although the current evidence is still mainly based on *in vitro* studies. Lactic acid is able to inactivate a wide range of reproductive tract pathogens, including *C. trachomatis*^22^ and HIV-1^37^. In the present study we investigated whether the anti-*Chlamydia* activity of lactic acid is merely associated with the pH, or other mechanisms may be involved. Lactic acid, at the concentrations found in the lactobacilli supernatants, was able to strongly inhibit EBs infectivity only at acid pH for all exposure times, differently from hydrochloric acid that did not show any activity in the same experimental conditions. These results indicate that a high concentration of H^+^ ions is necessary but not sufficient to inhibit *Chlamydia* EBs. Therefore, the presence of lactate in an acidic environment seems to be crucial for the activity. It remains to elucidate the specific mechanisms by which lactate moiety inactivates chlamydial EBs. Notably, the inhibitory activity exerted by the majority of *Lactobacillus* supernatants, characterized by similar lactate concentrations and pH values, was higher than that exerted by lactic acid solution, suggesting that other metabolites present in lactobacilli supernatants could determine a synergistic effect. Because *L. crispatus* strains were found to be the most active in counteracting *Chlamydia* infection, we assume that the effect of lactic acid may be enhanced by the pool of metabolites especially produced by this species.

Given the importance of the metabolic component in determining the inhibition of *Chlamydia*, we studied by ^1^H-NMR the profile of metabolites secreted by the vaginal lactobacilli. Metabolic variance was strictly correlated with the inhibitory activity, confirming the excellent anti-chlamydial profile of the majority of *L. crispatus* strains. By considering the median values of lactobacilli grouped according to anti-*Chlamydia* activity, major differences in the metabolomes were observed between the strains with low activity compared to the strains with high or intermediate activity, which in turn appeared more similar to each other.

Interestingly, the metabolomic analysis highlighted the increased consumption of glucose by the most active strains. We demonstrated that glucose depletion represents an additional mechanism of action for lactobacilli antagonism towards *Chlamydia*. Chlamydial EBs, adapted for extracellular survival and primed for infection of susceptible host cells, have historically been described as metabolically dormant. On the other hand, it has been recently reported that *C. trachomatis* EBs have the capacity for considerable metabolic and biosynthetic activity and utilize glucose as an energy source^38^ to fuel the EB to RB developmental transition^39^. Glucose fermentation in lactobacilli leads to the formation of organic acids, including lactic acid. Therefore, consumption of glucose and production of organic acids are metabolically interrelated and represent defensive strategies implemented by vaginal lactobacilli against the attack of pathogens, such as *Chlamydia*. Notably, an increase of glucose concentration in the vaginal fluids collected from women affected by bacterial vaginosis was reported in a recent investigation^40^. These findings can be interpreted by assuming that the lack of lactobacilli, and mainly of those strains with high efficiencies of glucose fermentation, is associated with a greater availability of glucose in the vaginal environment. We hypothesize that the availability of glucose, in turn, could promote growth of undesirable microorganisms, including bacteria responsible for bacterial vaginosis and chlamydiae.

In conclusion, the present *in vitro* study demonstrates the ability of different *Lactobacillus* strains of vaginal origin to inactivate *C. trachomatis* EBs through the production of extracellular metabolites in an acidic environment. In particular, we found that high concentrations of *L. crispatus* inhibit *C. trachomatis* infectivity *in vitro*, stressing once again the importance of this species for the vaginal health. Because*C. trachomatis* is an intracellular organism, further studies will be needed to investigate the interactions between *C. trachomatis*-lactobacilli-epithelial cells.

## Methods

### *Lactobacillus* culture conditions

Lactobacilli were cultured in de Man, Rogosa and Sharpe (MRS) broth (Difco, Detroit, MI) supplemented with 0.05% L-cysteine. Incubation was carried out in anaerobic jars supplemented with Anaerocult C (Merck, Milan, Italy) for 18 h at 37 °C.

### Preparation of lactobacilli fractions

The turbidity of 18-h lactobacilli cultures was measured by a Beckman spectrophotometer (Beckman Coulter, Fullertone, CA), considering that an optical density (OD_600_) of 0.4 corresponds to a cell concentration of 10^8^ colony forming unit (CFU)/mL. Lactobacilli cultures were adjusted to an OD_600_ of 2.0 with sterile MRS (cell concentration: 5 × 10^8 ^CFU/mL) and centrifuged at 5,000 × g for 10 min at 4 °C. Supernatants were filtered through a 0.2 μm membrane filter to obtain stock cell free supernatants (CFS). Ten-fold dilutions (1:10 and 1:100) of the stock CFS were prepared in sterile saline. Cell pellets (CP) were washed and resuspended in sterile saline to obtain stock suspensions of 5 × 10^8 ^CFU/mL.

### *C. trachomatis* propagation and preparation of EBs

*C. trachomatis* strain GO/86, serotype D, was used in this study^41^,^42^. This strain was clinically isolated in 1986 from an urethral swab submitted to the Microbiology Laboratory of Sant’Orsola-Malpighi University Hospital, Bologna, for routine diagnostic procedures and belongs to the laboratory collection. *C. trachomatis* was propagated in HeLa cells, cultured in Dulbecco’s minimal essential medium (DMEM), supplemented with 10% foetal bovine serum, 1% L-glutamine 200 mM, and antibiotics (vancomycin 10 mg/L, gentamicin 10 mg/L and amphotericin B 0.3 mg/L). For the preparation of EBs, confluent HeLa cells were infected with *Chlamydia* in DMEM medium supplemented with cycloheximide 1 μg/mL, centrifuged at 640 × g for 2 hours to facilitate cell penetration, then incubated at 37 °C with 5% CO_2_ for 48 hours^23^. HeLa cells were then detached and fragmented by sonication, by using a Bandelin sonicator at minimum power. Samples were centrifuged at 500 × g for 10 minutes at 4 °C, and supernatants, which contain EBs, were further centrifuged at 40,000 × g at 4 °C for 1 hour. The resulting pellets, containing the purified EBs, were resuspended in sucrose-phosphate-glutamate (SPG) 0.2 M, divided into small aliquots and stored at −70 °C.

### *C. trachomatis* inhibition test

Inhibition experiments with lactobacilli supernatants were carried out with three dilutions of the stock CSF (1:1; 1:10; 1:100). Inhibition experiments with lactobacilli cells were carried out with three different CP concentrations (2.5 × 10^8^, 2.5 × 10^7^ and 2.5 × 10^6^ CFU/mL). Lactobacilli CFS (100 μL of 1:1, 1:10, 1:100 dilutions) and CP (100 μL, 10 μL, 1 μL of stock suspension) were mixed with 5 × 10^3^ IFU of EBs and diluted to 200 μL with sterile phosphate buffered saline. pH values were measured in the final volume. The same amount of purified EBs was used as control after verifying the lack of effects exerted by MRS medium on EBs infectivity. Mixes were incubated for 7, 15 and 60 minutes at 37 °C in 5% CO_2_ atmosphere, afterward they were centrifuged at 20,000 × g for 10 minutes at 4 °C. Supernatants were used to infect HeLa cells, grown to confluence in individual tubes containing sterile coverslips, and centrifuged at 640 × g for 2 hours to facilitate cell penetration, then incubated at 37 °C with 5% CO_2_ for 48 hours. Inhibition experiments were also carried out with lactic acid and hydrochloric acid solutions at different concentrations (10 mM and 50 mM) and pH values (pH 4 and 7), in order to evaluate the effect of pH and organic/inorganic acids to *C. trachomatis* infectivity. In addition, orotic acid was tested at the concentrations of 30 μM in order to verify the ability of this molecule to inhibit *Chlamydia* infectivity. Orotic acid 30 μM was also tested in combination with lactic acid (10/50 mM, pH 4/7), to evaluate a possible synergic effect with lactic acid. In order to address the mechanistic question of the importance of glucose depletion in the inhibition of *Chlamydia* infectivity, supernatants of *L. crispatus* BC1 and *L. gasseri* BC13 (dilution 1:1) were added with glucose 30 mM and used in the inhibition experiments.

*C. trachomatis* infection was evaluated by counting *Chlamydia* IFU by direct immunofluorescence, using a monoclonal antibody against the chlamydial membrane lipopolysaccharide antigen conjugated with fluorescein (Meridian, Cincinnati, OH, USA), as previously reported^42^. Slides were observed under epi-fluorescence microscope (Eclipse E600, Nikon, Japan) equipped with a super high pressure mercury lamp and Plan Fluor DLL 20×, 40×, 100× lenses. The number of IFU was counted in 30 randomly chosen 200× microscopic fields.

### ^1^H-NMR analysis of metabolic profiles of lactobacilli supernatants

One ml of CFS obtained from lactobacilli was added to 160 μl of a D_2_O solution of 3-(trimethylsilyl)-propionic-2,2,3,3-d4 acid sodium salt (TSP) 6.25 mM set to pH 7.0 by means of a 100 mM phosphate buffer. ^1^H-NMR spectra were recorded at 298 K with an AVANCE III spectrometer (Bruker, Milan, Italy) operating at a frequency of 600.13 MHz, following the procedure previously described^40^,^43^. The signals were assigned by comparing their chemical shift and multiplicity with Chenomx software data bank (Chenomx Inc., Canada, ver 8.02). In order to search for correlations between anti-*Chlamydia* activity and metabolome of lactobacilli, a PCA model was built on the concentration changes of the identified molecules, scaled to unit variance. The PCA algorithm calculates and sorts linear combinations of the original variables, so to highlight the data structure by means of a low number of orthogonal projections (Principal Components). The score-plot is the representation of the samples in the generated space and highlights the similarities and differences between the samples. For each molecule, the correlation between the concentration change and the loading value with the component of PCA describing a metabolome-activity link was calculated. When the correlation was higher than 0.6, a statistically significant difference between groups H and L was searched by means of a Wilcoxon test, with an accepted Bonferroni-adjusted P value of 0.05.

### Statistical analysis

All statistical analysis were performed by using R computational software (www.R-project.org), applying the non-parametric signed- or matched paired- Wilcoxon rank tests. 1- or 2-tailed tests were used as specified in the manuscript. Differences were deemed significant for P values < 0.05. Spearman correlation was calculated by using GraphPad software (GraphPad software Inc., San Diego, CA).

## Additional Information

**How to cite this article**: Nardini, P. *et al.* *Lactobacillus crispatus* inhibits the infectivity of *Chlamydia trachomatis* elementary bodies, in vitro study. *Sci. Rep.* **6**, 29024; doi: 10.1038/srep29024 (2016).

## Supplementary Material

Supplementary Information

## Figures and Tables

**Table 1:** Vaginal lactobacilli used in the present study.

Species	Strain	Accession n.
*L. crispatus*	BC1	AB976542
*L. crispatus*	BC2	AB976543
*L. crispatus*	BC3	AB976544
*L. crispatus*	BC4	AB976545
*L. crispatus*	BC5	AB976546
*L. crispatus*	BC6	AB976547
*L. crispatus*	BC7	AB976548
*L. crispatus*	BC8	AB976549
*L. gasseri*	BC9	AB976550
*L. gasseri*	BC10	AB976551
*L. gasseri*	BC11	AB976552
*L. gasseri*	BC12	AB976553
*L. gasseri*	BC13	AB976554
*L. gasseri*	BC14	AB976555
*L. vaginalis*	BC15	AB976556
*L. vaginalis*	BC16	AB976557
*L. vaginalis*	BC17	AB976558
