# Supplementary material for: Lactobacillus crispatus inhibits the infectivity of Chlamydia trachomatis elementary bodies, in vitro study
Source: Sci Rep. 2016 Jun 29;6:29024. doi: 10.1038/srep29024 (PMC4926251; doi:10.1038/srep29024)

Lactobacillus crispatus inhibits the infectivity of *Chlamydia trachomatis* elementary bodies, in vitro study

Paola Nardini, Rogers Alberto Ñahui Palomino, Carola Parolin, Luca Laghi, Claudio Foschi, Roberto Cevenini, Beatrice Vitali & Antonella Marangoni

Supplementary Table S1. pH values of *Lactobacillus* supernatants

Strain	pH values		
	2.5×10^8 CFU/mL	2.5×10^7 CFU/mL	2.5×10^6 CFU/mL
BC1	4.13	6.21	6.85
BC2	3.88	5.15	6.76
BC3	4.04	6.09	7.03
BC4	3.9	6.22	6.97
BC5	3.79	6.34	7.05
BC6	3.71	4.30	6.97
BC7	3.85	4.45	7.05
BC8	4.25	4.82	7.08
BC9	3.80	4.74	7.13
BC10	4.86	7.15	7.31
BC11	4.40	6.11	7.15
BC12	4.19	5.93	7.15
BC13	4.45	6.47	7.06
BC14	4.14	6.81	7.27
BC15	4.04	6.64	7.23
BC16	4.55	5.47	7.04
BC17	5.28	6.32	7.27

Supplementary Table S2. Concentrations of lactate, orotate, phenylalanine, isoleucine, valine, tyrosine glucose and tryptophan in cell free supernatants of vaginal lactobacilli. Concentrations were calculated by ^{1}H-NMR as differences from MRS medium. Values are expressed as mmol/l. Significant differences between supernatants and MRS were indicated with an asterisk (P<0.05, 2-tailed Wilcoxon signed rank test)

Strain	Lactate*	Orotate*	Phenylalanine	Isoleucine*	Valine*	Tyrosine*	Glucose*	Tryptophan*
**BC1**	2.910	0.029	-0.429	0.409	0.549	0.026	-28.500	-0.147
**BC2**	6.830	0.030	-0.375	0.380	0.509	0.413	-20.900	-0.172
**BC3**	9.450	0.007	0.129	1.100	0.890	0.396	-19.000	-0.652
**BC4**	3.320	0.029	0.172	0.231	0.351	0.469	-24.000	-0.024
**BC5**	5.100	0.017	0.157	0.244	0.334	0.112	-19.500	-0.068
**BC6**	7.870	0.022	0.126	0.225	0.258	0.132	-19.200	-0.196
**BC7**	1.420	0.016	-0.041	0.737	0.628	0.244	-23.600	-0.118
**BC8**	3.050	0.023	0.033	0.908	0.838	0.234	-19.700	-0.300
**BC9**	4.750	0.007	-0.294	0.344	0.550	0.026	-26.700	-0.181
**BC10**	9.400	0.005	0.745	0.519	0.571	0.627	-6.110	-0.209
**BC11**	14.600	0.003	0.641	0.632	0.691	0.618	-18.100	-0.245
**BC12**	9.470	0.001	0.521	0.770	0.825	0.531	-21.400	-0.238
**BC13**	1.620	0.008	-0.178	0.584	0.892	0.701	-28.800	-0.101
**BC14**	1.940	0.007	2.390	1.810	2.040	0.664	-20.200	-0.370
**BC15**	47.400	0.027	-1.300	0.432	0.068	-0.221	-26.400	-0.092

**BC16**	24.400	0.003	-0.948	0.827	0.687	0.453	-16.400	-0.596
**BC17**	23.400	0.009	0.180	0.950	0.761	0.096	-20.800	-0.283

Supplementary Table S3. P-values calculated on cell free supernatant median values by 1-tailed Wilcoxon signed rank test vs control

Strain	P – value
BC1	0.1170
BC2	0.0210
BC3	0.6393
BC4	0.0953
BC5	0.2761
BC6	0.0057
BC7	0.1160
BC8	0.0611
BC9	0.3172
BC10	0.9980
BC11	0.4551
BC12	0.7616
BC13	0.0774
BC14	0.6824
BC15	0.5940
BC16	0.3262
BC17	0.8984

Supplementary Table S4. Increase of *C. trachomatis* infectivity following the addition of glucose to cell free supernatants of *L. crispatus* BC1 and *L. gasseri* BC13. Dilutions 1:1 of *L. crispatus* BC1 and *L. gasseri* BC13 supernatants were tested. Increase was calculated as ratio between the infectivity of the supernatant added with glucose 30 mM and the infectivity of the corresponding not enriched supernatant. Significant increases were indicated with an asterisk (P<0.05, 1-tailed Wilcoxon matched paired rank test)

**Strain**	**Contact time**		
	**7 minutes**	**15 minutes**	**60 minutes**
L. crispatus BC1	51.2*	-	-
L. gasseri BC13	8.7*	6.1*	-

-: no variation

Supplementary Figure S1. Effect of orotic acid and combinations of orotic acid/lactic acid on *C. trachomatis* infectivity. Experiments were performed with 30 µM orotic acid (a) and orotic acid (30 µM) in combination with lactic acid at different concentrations (10 mM and 50 mM) and pH values (4 and 7)(b). *C. trachomatis* infectivity was evaluated at different and time points [7 minutes (white bars), 15 minutes (grey bars) and 60 minutes (black bars)], as number of IFU/microscopic field. The results were expressed in percentage compared with control, taken as 100% (dotted bars). Bars represent median values, error bars represent median absolute deviations. Statistical significance was calculated vs control. * P < 0.05

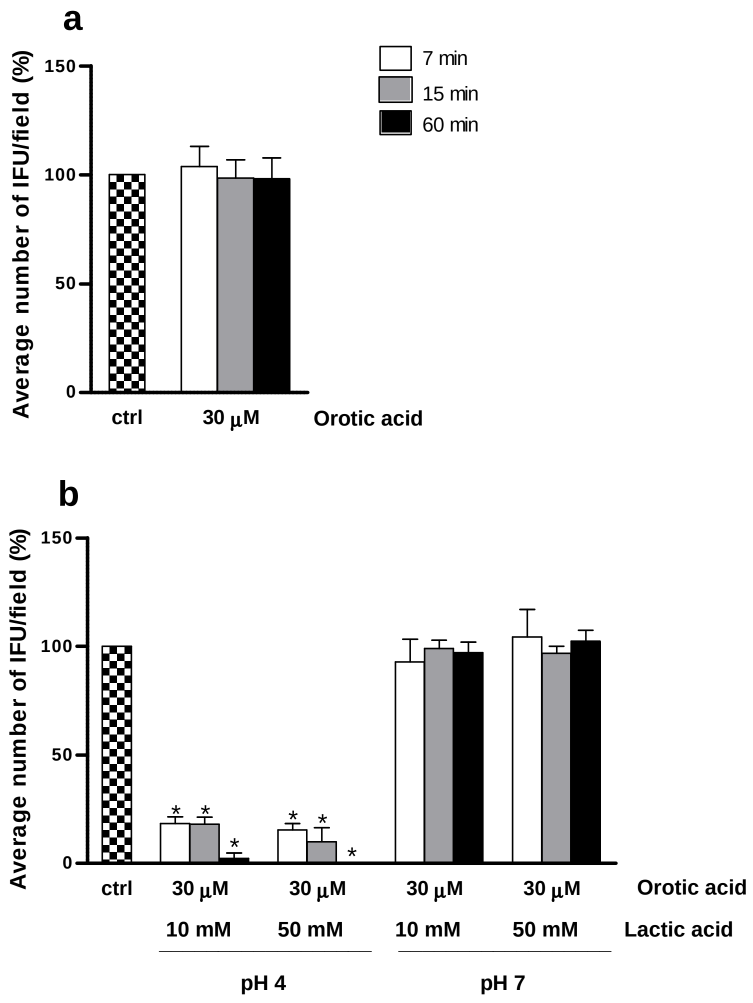

Supplement: Supplementary Information [file srep29024-s1.pdf]
